# Niobium Tungsten Oxide in a Green Water-in-Salt Electrolyte Enables Ultra-Stable Aqueous Lithium-Ion Capacitors

**DOI:** 10.1007/s40820-020-00508-z

**Published:** 2020-08-18

**Authors:** Shengyang Dong, Yi Wang, Chenglong Chen, Laifa Shen, Xiaogang Zhang

**Affiliations:** 1 School of Chemistry and Materials Science, Institute of Advanced Materials and Flexible Electronics (IAMFE), Nanjing University of Information Science and Technology, Nanjing, 210044 People’s Republic of China; 2 Jiangsu Key Laboratory of Electrochemical Energy Storage Technologies, College of Materials Science and Technology, Nanjing University of Aeronautics and Astronautics, Nanjing, 210016 People’s Republic of China; 3 Max Planck Institute for Solid State Research, Heisenbergstrasse 1, 70569 Stuttgart, Germany

**Keywords:** Aqueous hybrid capacitors, Water-in-salt electrolyte, Niobium tungsten oxide, Ultra-stability, High power density

## Abstract

Aqueous hybrid supercapacitors are attracting increasing attention due to their potential low cost, high safety and eco-friendliness. However, the narrow operating potential window of aqueous electrolyte and the lack of suitable negative electrode materials seriously hinder its future applications. Here, we explore high concentrated lithium acetate with high ionic conductivity of 65.5 mS cm^−1^ as a green “water-in-salt” electrolyte, providing wide voltage window up to 2.8 V. It facilitates the reversible function of niobium tungsten oxide, Nb_18_W_16_O_93_, that otherwise only operations in organic electrolytes previously. The Nb_18_W_16_O_93_ with lithium-ion intercalation pseudocapacitive behavior exhibits excellent rate performance, high areal capacity, and ultra-long cycling stability. An aqueous lithium-ion hybrid capacitor is developed by using Nb_18_W_16_O_93_ as negative electrode combined with graphene as positive electrode in lithium acetate-based “water-in-salt” electrolyte, delivering a high energy density of 41.9 W kg^−1^, high power density of 20,000 W kg^−1^ and unexceptionable stability of 50,000 cycles.

**Electronic supplementary material:**

The online version of this article (10.1007/s40820-020-00508-z) contains supplementary material, which is available to authorized users.

## Highlights

- A green water-in-salt electrolyte was developed using lithium acetate as solute with a wide electrochemical stability window of 2.8 V.
- Molecular dynamics simulation confirmed the nature of water-in-salt electrolyte, where hydrogen bonds of water–water were disrupted and ionic interactions became stronger than dilute solution.
- Nb_18_W_16_O_93_-based lithium-ion capacitors delivered unexceptionable stability over 50,000 cycles.

**Electronic supplementary material:**

The online version of this article (10.1007/s40820-020-00508-z) contains supplementary material, which is available to authorized users.

## Graphical abstract

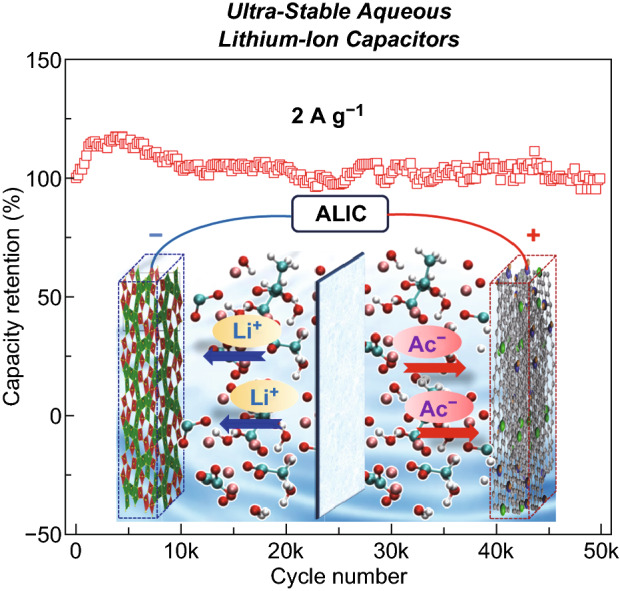

## Introduction

There exist tremendous needs for grid storage to couple the renewable-but-intermittent solar and wind energy [1–3]. Electrochemical energy storage is promising, because it provides a modular solution, which is less geographically restricted than pumped hydro- and compressed air storage, etc. [4–6]. The metric to evaluate energy storage is the levelized cost and the life time of the devices. To minimize such cost, one should use aqueous electrolytes to lower the acquisition cost and maximize the cycle life so that maintenance cost can be low [7–10]. Besides, compared with organic cells, aqueous electrolytes are noninflammable and eco-friendly. Therefore, it is strategically advantageous to employ electrochemical devices with aqueous electrolytes for large-scale energy storage.

Among the aqueous energy storage technologies, there are electrochemical capacitors (ECs) and rechargeable batteries, etc. The advantages of ECs are the ultra-long cycle life and the high power performance [11, 12]. However, ECs suffer from low energy density, usually less than 10 Wh kg^−1^, and their self-discharge is quite severe, particular for the aqueous ECs [13]. Rechargeable batteries, especially lithium-ion batteries (LIBs), provide high energy density [14, 15]. But the power character and cycling lifespan are unacceptable [16, 17]. There are also hybrid energy storage devices, for example, lithium-ion hybrid capacitors (LICs), where one electrode operates on Faradaic reactions, and the other uses electrical double layer (EDL) storage [18–20]. For example, during charge, the positive electrode incorporates anions adsorption and the negative electrode takes in cations in reductive ion insertion; during discharge, the ion migration direction is reversed. The trade-off between ECs and LIBs of LICs recently received much attention due to their excellent energy–power characters and long lifespan [21–23]. However, the state-of-the-art aqueous lithium-ion hybrid capacitors (ALICs) have been limited with their narrow electrochemical stability window of the water (~ 1.23 V) and sluggish diffusion in the bulk of battery-type materials.

Fortunately, “water-in-salt” electrolytes (WiSEs) are effective strategies to extend the electrochemical stability window of aqueous electrolytes [24–28]. This approach was first reported by Suo et al. using 21 m lithium bis(trifluoromethanesulfonyl) (LiTFSI) [24]. Albeit with the attractive performance achieved, the majority of previous reports make use of highly concentrated fluorinated salt (such as LiTFSI, LiN(SO_2_C_2_F_5_)_2_) [25], which are notoriously wasteful. This goes against the original aims of aqueous cells, which are environmental friendliness and low cost. Recently, fluorine-free WiSEs received much attention due to their green and low-cost. Among them acetate (Ac)-based WiSEs are the famous alternatives, including potassium acetate (KAc) [29], KAc + lithium acetate (LiAc) [9], KAc + sodium acetate (NaAc) [30], and ammonium acetate (AmAc) [31], etc.

Another main challenge of ALICs is how to promote the quick-charge characteristic and high stability of battery-type materials. Recently, Grey et al. reported a niobium tungsten oxide − Nb_18_W_16_O_93_ (NbWO), which adopt bronze-like structure with high lithium-ion diffusion coefficient in the order of 10^−13^ m^2^ s^−1^ [32]. This material possesses high rate characteristic and cycle stability even the particle size is of the order of micrometers. However, the high potential of lithium-ion intercalation is not very ideal as a negative electrode material in organic cells.

Herein, we demonstrate the efficacy of high concentrated lithium acetate as a green, fluorine-free “water-in-salt” electrolyte with low viscosity, high ionic conductivity, and wide voltage window up to 2.8 V. The molecular dynamics (MD) simulation disclose that the interaction between H_2_O and Ac^−^ arises in the concentrated electrolyte to break the ubiquitous hydrogen bonding network between H_2_O molecules, which is mainly responsible for the expanded electrochemical stability window. Furthermore, a novel ALIC is developed, which employs Nb_18_W_16_O_93_ (NbWO) as a negative electrode and a positive electrode of oxygen-enriched crumpled graphene (OECG) in LiAc-based WiSE with high energy-power density and unexceptionable stability of 50,000 cycles.

## Experimental Section

### Preparation of Nb_18_W_16_O9_3_

Nb_18_W_16_O_93_ was prepared by a simple solid state reaction established in Grey’s previous work [32] with a minor modification. Typically, Nb_2_O_5_ and WO_3_ powers (Nb:W = 9:8 in mol/mol) were used as the raw materials to synthesize Nb_18_W_16_O_93_ (NbWO). After thoroughly grinding in an agate mortar with moderate absolute ethanol, the resulting mixture was pressed into pellets at a pressure of 10 MPa and then annealing in a Pt crucible covered with a Pt lid at 700 °C for 12 h with a heating rate of 10 °C min^−1^ followed by 1200 °C for 12 h with a heating rate of 5 °C min^−1^ in air atmosphere.

### Materials Characterization, Electrochemical Measurement and Molecular Dynamics Simulation

Detailed information about the materials characterization, electrochemical measurement, and molecular dynamics (MD) simulations are provided in the Supporting Information.

## Results and Discussion

### Characterization of Water-in-Salt Electrolyte

**Fig. 1:**
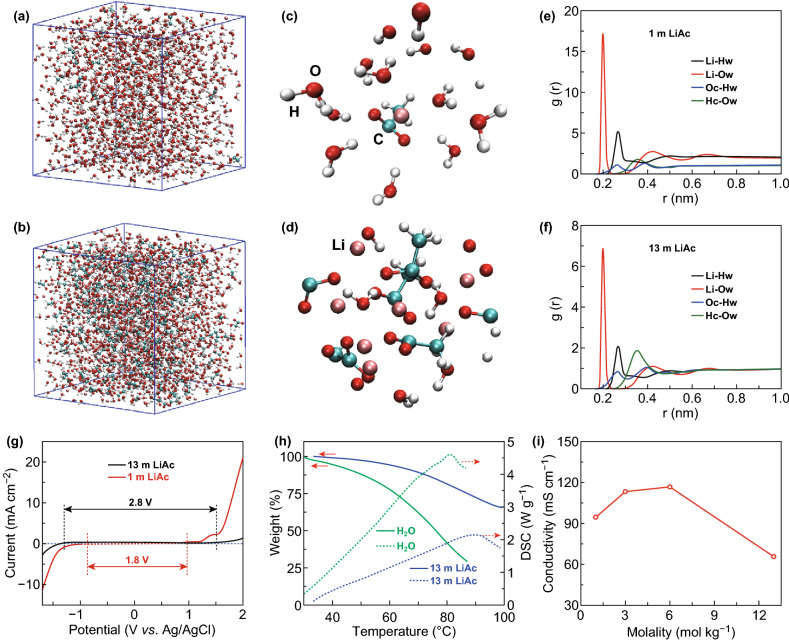
Physicochemical properties of the LiAc aqueous electrolytes. Snapshots of microstructure for the 1 m LiAc (**a, c**) and the 13 m LiAc electrolyte (**b, d**) by MD simulations after 4 ns. Atom colors: Li, brown; O, red; H, gray; C, blue. RDF curves of Li-Hw, Li-Ow, Oc-Hw, and Hc-Ow, respectively, in the 1 m LiAc (**e**) and the 13 m LiAc electrolyte (**f**). Hw represents H atom in H_2_O, Ow represents O atom in H_2_O, Oc represents O atom connected with C atom in Ac^−^, Hc represents H atom connected with C atom in Ac^−^. **g** Cyclic voltammetry (CV) curves collected in 1 m and 13 m LiAc at 10 mV s^−1^. **h** TG/DSC data of the H_2_O and the 13 m LiAc electrolyte. **i** The conductivity as a function of their molality values

Concentrated LiAc solutions achieve molalities up to 13 m, corresponding to about 4 H_2_O molecules per Li^+^. Molecular dynamics (MD) simulations were first performed to study the variations of the spatial micro-configurations of aqueous LiAc electrolyte system (Table S1). Figure 1a, b exhibit the snapshot of microstructure for 1 and 13 m LiAc solution. It can be found that, in 1 m LiAc, the Li^+^ freely coordinated with H_2_O molecules (Fig. 1c). The hydrogen bond is the main interaction in this dilute solution. However, in 13 m LiAc, Ac^−^, Li^+^, and H_2_O molecules are complete mixed together (Fig. 1d). The H_2_O molecules mainly interact with the nearby Li^+^ and Ac^−^, and the hydrogen bonds almost disappear. This will greatly affect the physical and chemical properties, such as density, viscosity, conductivity, thermostability, and electrochemical stability window [33]. As shown in the radial distribution function (RDF) curves (Fig. 1e, f), the distance between Ow (O atom in H_2_O) and Hc (H atom connected with C atom in Ac^−^) shifts from 0.36 (in 1 m LiAc) to 0.35 nm (in 13 m LiAc). This may be caused by the fact that the interaction between H_2_O molecules and Ac^−^ becomes stronger in the concentrated electrolyte. Besides, the peaks of Oc–Hw and Hc-Ow become stronger in the concentrated solution. This means that Ac^−^ tends to interact with the H_2_O molecules when the number of Ac^−^ increases. Table S2 summarizes the physical properties of 1 and 13 m LiAc electrolytes. MD predicted the densities of 1 and 13 m LiAc are 0.99 and 1.18 g cm^−3^, respectively. The viscosity of concentrated electrolyte is only 9.5 mPa s at 25 °C, which value is superior to the most previously reported WiSEs and organic electrolytes [9, 25, 34, 35].

The electrochemical stability window of LiAc-based electrolytes was evaluated via cyclic voltammetry (CV) at a scan rate of 10 mV s^−1^. As shown in Fig. 1g, it demonstrates that the 13 m LiAc delivers a wide electrochemical window of about 2.8 V using cutoff current density of 50 μA cm^−2^, from − 1.3 to 1.5 V (vs. Ag/AgCl). However, as for 1 m LiAc electrolyte, the stability window is only about 1.8 V. The thermogravimetric/differential scanning calorimetry (TG/DSC) data (Fig. 1h) also demonstrate that 13 m LiAc electrolyte has a much better thermostability than water. As shown in Fig. 1i, the LiAc electrolytes possess high conductivity of 65.5 mS cm^−1^ even at high molality of 13 mol kg^−1^, which exceeds that of the LiTFSI-based and hydrate-melt electrolytes at the comparable concentrations [24, 25].

### Characterization of Niobium Tungsten Oxide

**Fig. 2:**
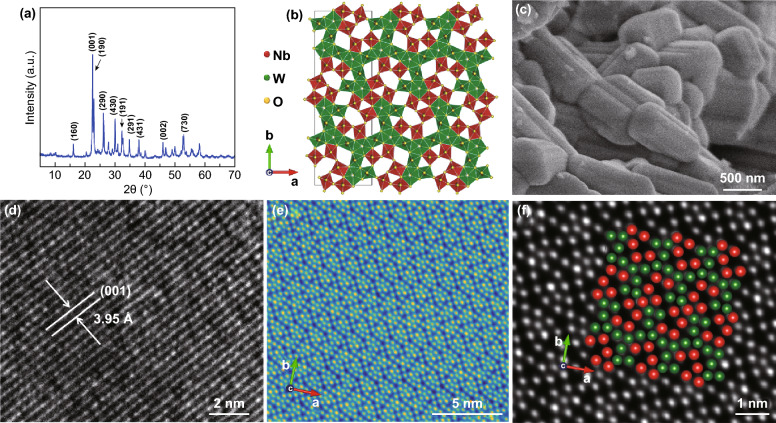
Microstructural characterizations of NbWO. **a** XRD pattern. **b** Structural diagram of NbWO along the *c* axis. **c** SEM image. **d** HR-TEM image. **e**, **f** Aberration-corrected HAADF–STEM images of atomic arrangement which match the inset structure model viewed along the [001] crystallographic direction, W:green; Nb: red

The as-prepared Nb_18_W_16_O_93_ were first characterized by X-ray diffraction (XRD) to determine its crystallographic structure. As can be seen in Fig. 2a, all of the diffraction peaks can be well indexed to the orthorhombic tetragonal tungsten bronze (JCPDS No 75-0561) [32, 36]. The sharp and well-recognized diffraction peaks demonstrates the high crystallinity arising from the high temperature annealing. As shown in the structural diagram in Fig. 1b, the superstructure of NbWO results from partial filling of pentagonal tunnels by –M–O– polyhedrons (M=Nb or W) to form pentagonal bi-pyramids, with the distorted octahedra of the tetragonal tungsten bronzes. We further investigated the microstructure of the as-prepared NbWO by scanning electron microscopy (SEM) and high-resolution transmission electron microscopy (HRTEM). In the SEM images (Figs. 2c and S1), NbWO shows large block morphology (1–2 μm primary, 10–20 μm agglomerate). Obviously, the crystal lattice fringes with the interplanar distance of 3.95 Å can be observed in Fig. 2d, which is consistent with the interplanar spacing of the (001) plane of NbWO. More important, the atomic structure of NbWO is revealed by aberration-corrected scanning transmission electron microscopy (STEM) with high-angle annular dark field (HAADF) detector for the first time. The corresponding HAADF images are presented in Fig. 2e, f, with the [001] crystallographic direction. Due to the much weaker electron scattering of O atoms than that of Nb and W, the O atoms are invisible in the HAADF image. The atomic arrangement in Fig. 2f matches well with the inset structure model as shown in Fig. 2b.

### Evaluation of Li-Ion Storage

**Fig. 3:**
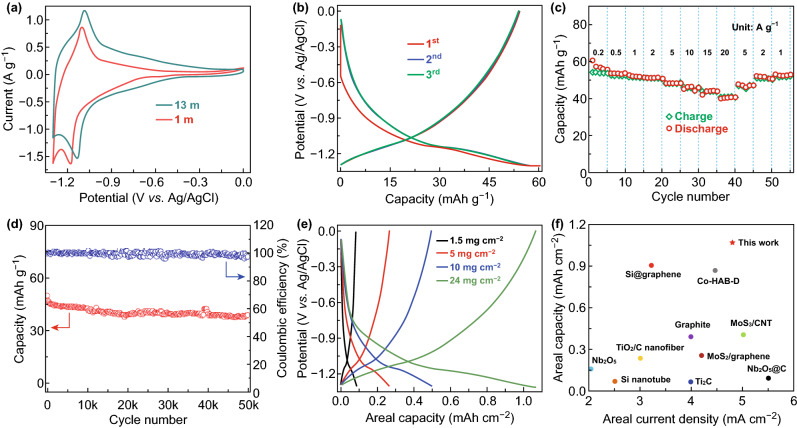
Lithium-ion storage performance of NbWO electrode. **a** Typical CV curves in 1 m and 13 m LiAc electrolytes at 2 mV s^−1^. **b** GCD curves at 200 mA g^−1^. **c** Rate performance. **d** Cycling stability at 2 A g^−1^. **e** GCD curves with various active mass loadings. **f** Areal capacity comparison of the NbWO electrode with representative anode materials for non-aqueous energy storage in previous reports

Reaction of NbWO with Li^+^ proceeds in the potential window of − 1.3 to 0 V (all potentials of three electrodes hereafter are referred to Ag/AgCl). Figure 3a exhibits the typical CV curves of NbWO electrode in 1and 13 m LiAc electrolytes. Apparently, a big polarization is observed in 1 m LiAc solution, which is attributed to the reduction of H_2_O with H_2_ evolution. On the contrary, a reversible redox pair appears at around − 1.1 V in 13 m LiAc solution, which corresponds to the reversible Li^+^ insertion/extraction reaction of NbWO. The small polarization potential of 0.08 V means superior high rate performance. Figure 3b shows the galvanostatic charge–discharge (GCD) curves of the NbWO electrode at 200 mA g^−1^ with a reversible capacity of about 54 mAh g^−1^ in 13 m LiAc electrolyte. However, in 1 m LiAc electrolyte, as shown in Fig. S2, the NbWO electrode doesn’t work at all with the same potential window. When the kinetics was examined over a range of current densities from 200 mA g^−1^ to 15 A g^−1^, NbWO showed superior rate performance (Fig. 3c) in 13 m LiAc electrolyte. Even elevate to 20 A g^−1^, the capacity of NbWO is still remain at about 40 mAh g^−1^, equivalent to 74.1% of the capacity at 200 mA g^−1^. The cycling performance is one of the longest cycling performances for batteries in previous reports. The capacity retention is up to 85.5% after 50,000 cycles at 2 A g^−1^ (Fig. 3d). In order to increase the areal capacity, we further investigated the electrochemical characteristics of NbWO electrodes with high mass loading from 1.5 to 24 mg cm^−2^, which is one of the key indicators for practical applications. As shown in Fig. 3e, the areal capacity can be increased almost linearly from 0.08 to 1.09 mAh cm^−2^. More significantly, 85.2% of specific capacity of the 1.5 mg cm^−2^ electrode is still exhibited even in the 16 times thicker electrode (24 mg cm^−2^), which confirms prominent transport kinetics in the NbWO electrode. We compared the areal performance of NbWO with other secondary batteries, including LIBs and Na-ion batteries (NIBs) even in organic systems (Fig. 3f). Obviously, among the previous reported graphite [37], Si [38, 39], metal oxides/sulfides/carbides [40–45], even the emerging conductive metal organic framework (MOF) anodes [46], NbWO demonstrated one of the best integrative areal electrochemical character. The impressive electrochemical performance of NbWO may benefit from the rapid electrode kinetics, which is derived from its prop open framework network, and the high-stable LiAc-based WiSE.

We further conducted X-ray photoelectron spectroscopy (XPS) at different states of charge (SOC) to analyze the valence of niobium and tungsten (Fig. S3). As shown in Fig. S3b, the Nb 3*d* binding energies of NbWO are located at 210.3 and 207.6 eV, confirming Nb(V); when discharged to − 1.3 V (all potentials are vs. Ag/AgCl), the two sharp peaks at 209.8 and 207.1 eV are indexed to Nb(IV) [47]. Upon charging back to 0 V, the Nb 3*d* peaks recovered back to the original binding energies of Nb(V). Similarly, W also shows reversible redox based on W(VI)/W(V) during charge/discharge process (Fig. S3c). In addition, with a careful observation, there appears to be a larger shift in the binding energies of W(4f) for the discharged to − 1.0 V, indicating a slight tendency for W reduction initially. To reveal the impact of the Li-ion storage on the structure of NbWO, we collected ex situ XRD patterns at a selected SOC in the first cycle. As shown in Fig. S4, the main peaks minor shift to lower degree after lithiation. Upon de-lithiation to 0 V, the main peaks return back to their initial positions gradually. It indicates that NbWO has excellent structural stability during the electrochemical process.

**Fig. 4:**
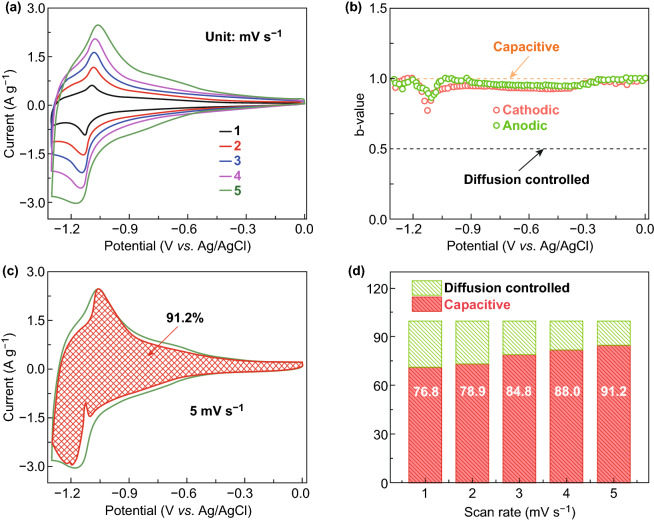
Kinetic analysis of NbWO electrode. **a** CV curves at various scan rates from 1 to 5 mV s^−1^. **b** Determination of the *b*-values at various potential. Contribution of the capacitive behavior at 5 mV s^−1^ (**c**) and 1 to 5 mV s^−1^ (**d**)

Kinetic analysis using CV technology was conducted to acquire further insight into the excellent electrochemical character of NbWO. Figure 4a displays the typical CV curves for the NbWO electrode at scan rates from 1 to 5 mV s^−1^. It is important to highlight that the small potential offsets are nearly constant even the scan rate increases to 5 mV s^−1^, demonstrating small polarization even at high rates. Indeed, the current in CV curves can be deconvoluted to two components: the capacitive non-diffusion-controlled process and the diffusion-controlled process. To shed light on the charge storage kinetics, we analyze the response current (*i*) by the power-law dependence, *i* = *av*^*b*^, where *v* is the scan rate in CV, and *a* and *b* are coefficients. Particularly, if the redox reaction is diffusion controlled*, b* is 0.5; *b* is 1.0 when non-diffusion controlled. As shown in Fig. 4b, all the *b*-values are between 0.75 and 1, indicating that the current is predominantly capacitive even at redox region.

The kinetics can also be quantified by deconvoluting the current, *i*, at a particular potential *V* into capacitive (*k*_1_*v*) and semi-infinite linear diffusion-controlled processes (*k*_2_*v*^1/2^) [40]:

1 $$i\left( V \right) = k_{1} v + k_{2} v^{1/2}$$

Under this mathematical treatment, the extent of capacitive storage of NbWO is about 91.2% at 5 mV s^−1^ (Fig. 4c), which is presented by the red-shaded area. The quantified results (Fig. 4d) show that the capacitive capacity is improved gradually with increasing scan rates. The value of 76.8% obtained at 1 mV s^−1^ finally reaches the value of 91.2% at 5 mV s^−1^.

**Fig. 5:**
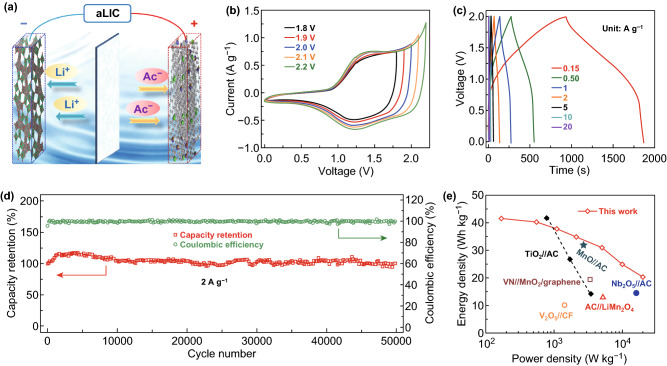
Electrochemical evaluation of (+)OECG//NbWO(−) ALIC. **a** The diagrammatic sketch of electrochemical reaction mechanism. **b** CV curves of the ALICs collected at different voltage windows at fixed 5 mV s^−1^. **c** GCD curves at different current density between 0 and 2 V. **d** Capacity retention and Coulombic efficiency for 50,000 cycles at a current density of 2 A g^−1^. **e** Ragone plots for the OECG//NbWO ALICs and compared with other aqueous and non-aqueous LICs reported in the studies

Considering its high rate capability, long cycle stability, NbWO represents a superb negative electrode material for ultra-stable ALICs. Figure 5a shows the ALIC’s configuration based on the NbWO negative electrode and oxygen-enriched crumpled graphene (OECG) positive electrode. Considering the electrochemical performance of OECG as shown in Figs. S5 and S6, the maximum work voltage of ALIC is forecasted to be 2.2 V with an optimized mass ratio of 1:1.1 (NbWO:OECG). A series of CV measurements with various voltage windows was conducted to identify the optimum working window (Fig. 5b). With an increase in the voltage to 2.0 V, the response current increased. However, when the ALIC is measured at a higher voltage of 2.1 or even 2.2 V, obvious polarization is observed. Thus, we use an optimum voltage window of 0–2.0 V to further evaluate the electrochemical performance of ALIC. As shown in Fig. 5c, the GCD curves for this ALIC at various current densities from 0.15 to 20 A g^−1^ exhibit symmetric quasi-triangular shapes, demonstrating a combination of different charge storage mechanisms of ECs and batteries. Specially, the GCD curves of the NbWO negative electrode and OECG positive electrode vs. Ag/AgCl reference electrode, along with the voltage profile of the ALIC are shown in Fig. S7. During the electrochemical reaction of positive electrode, there is a linear increase/decrease in potential with respect to time, which suggests the good absorption/desorption of Ac^−^ and subsequent double layer formation across the OECG electrode/electrolyte interface. The NbWO negative electrode has obvious slops in the potential region of − 1.3 to 0 V. Furthermore, it can also be observed that the OECG//NbWO exhibits a quasi-triangular voltage profile from 0 to 2 V, resulting from the difference between the positive electrode and the negative electrode. The ultra-long cycling stability of the ALIC (Fig. 5d) was demonstrated at a current density of 2 A g^−1^, with a capacity retention of almost 100% up to 50,000 cycles, which is one of the longest cycling performances for aqueous [48–50], and non-aqueous LICs [41, 45, 51–53] (see Table S3 for detail). In addition, the Ragone plot (Fig. 5e) demonstrates superior power and energy characteristics of the designed ALIC. A high energy density of 41.9 Wh kg^−1^ (based on the total active mass of both electrodes; similarly hereinafter) is achieved at the power density of 170.6 W kg^−1^. Even at a high power density of 20 kW kg^−1^, the ALIC still possesses an energy density of 20.2 Wh kg^−1^. Compared with other reported LICs in aqueous and non-aqueous systems, such as, AC//LiMn_2_O_4_ [54], V_2_O_5_//CF [48], VN//MnO_2_/graphene [55], Nb_2_O_5_//AC [38], TiO_2_//AC [56], MnO//AC [57], the OECG//NbWO ALIC device has superior energy and power characteristics. These results revealed that NbWO material effectively overcome the mismatch of charge storage and sluggish kinetics.

## Conclusions

In summary, we developed a green LiAc-based WiSE with a wide electrochemical stability window of 2.8 V. MD simulations confirmed the nature of LiAc-based WiSE system, where hydrogen bonds of water–water are disrupted and ionic interactions became stronger than dilute solution. Typical non-aqueous anode material of LIBs, Nb_18_W_16_O_93_, shows fully reversible lithium-ion storage in this green WiSE. Due to NbWO’s unexceptionable performance in micro-size particles and wide electrochemical window of green LiAc-based WiSE, NbWO-based ALIC delivers high energy/power characters and ultra-stable cyclability. After 50,000 cycles, the capacity retention is up to 100%, which is one of the longest cycling performances for hybrid capacitors. Besides, the cost of maintenance and recycling of LiAc-based hybrid capacitors is low. We believe that presented here green fluorine-free WiSE approach can make aqueous lithium-ion hybrid capacitors close to large-scale energy storage.

## Electronic supplementary material

Below is the link to the electronic supplementary material.

Supplementary material 1 (PDF 872 kb)
