# Supplementary material for: Niobium Tungsten Oxide in a Green Water-in-Salt Electrolyte Enables Ultra-Stable Aqueous Lithium-Ion Capacitors
Source: Nanomicro Lett. 2020 Aug 18;12:168. doi: 10.1007/s40820-020-00508-z (PMC7770661; doi:10.1007/s40820-020-00508-z)
Supplement: Supplementary file 1 — Supplementary material 1 (PDF 872 kb) [file 40820_2020_508_MOESM1_ESM.pdf]

Supporting Information for

Niobium Tungsten Oxide in a Green Water-In-Salt Electrolyte Enables Ultra-Stable Aqueous Lithium-Ion Capacitors

Shengyang Dong[1, 2], Yi Wang[3], Chenglong Chen[2], Laifa Shen[2, *], Xiaogang Zhang[2, *]

[1]School of Chemistry and Materials Science, Institute of Advanced Materials and Flexible Electronics (IAMFE), Nanjing University of Information Science & Technology, Nanjing 210044, People's Republic of China

[2]Jiangsu Key Laboratory of Electrochemical Energy Storage Technologies, College of Materials Science and Technology, Nanjing University of Aeronautics and Astronautics, Nanjing, 210016, People's Republic of China

[3]Max Planck Institute for Solid State Research, Heisenbergstrasse 1, Stuttgart 70569, Germany

*Corresponding authors. E-mail: azhangxg@nuaa.edu.cn (Xiaogang Zhang); lfshen@nuaa.edu.cn (Laifa Shen)

S1 Experimental Section

S1.1 Preparation of Oxygen-enriched Crumpled Graphene

Oxygen-enriched crumpled graphene (OECG) was synthesized according to our previous report [S1].

S1.2 Materials Characterization

The crystal structures of the obtained samples were characterized by XRD (PANalytical Empyrean) with Cu Kα radiation (λ = 1.54056 Å). The microscopic morphologies were characterized using field-emission scanning electron microscopy (FE-SEM, Hitachi S-4800) and transmission electron microscopy (TEM, JEOL JEM-ARM300F). The X-ray photoelectron spectroscopy (XPS) data were collected on a PerkinElmer spectrometer (PHI 550) with the Al-Kα (1486.6 eV) as X-ray source. Thermogravimetric/differential scanning calorimetry (TG/DSC) data were collected on a NETZSCH (STA 409 PC) thermal analyzer under air atmosphere at a temperature ramp of 5 °C min^{-1}.

S1.3 Electrochemical Measurement

All the half-cell tests were performed in three-electrode Swagelok cell setup, where free-standing activated carbon (AC) films were employed as a counter electrode and Ag/AgCl electrode (Sat. KCl) served as the reference electrode. AC was purchased by

Kuraray™. AC counter electrodes were prepared by mixing AC, Ketjen Black and PTFE (mass ratio of 8:1:1), with a mass loading of about 20 mg cm^{-2}. The aqueous 13 m LiAc were employed as the electrolytes. The OECG working electrode was prepared by mixing OECG, Ketjen Black, and carboxymethylcellulose (CMC)/styrene–butadiene rubber (SBR) (CMC/SBR = 2:1) binder with a mass ratio of 8:1:1 in water, where the homogeneous slurry was then casted onto the carbon fiber paper (CFP), followed by drying at 60 °C for 12 h under vacuum. NbWO electrode was prepared by mixing NbWO, CNT, and CMC/SBR with a mass ratio of 8:1:1. It is worthwhile mentioning that NbWO and CNT (mass ratio = 8:1) were first ball-milled with moderate absolute ethanol at 400 rpm for 2 h. The obtained powder was then dried in air at 80 °C overnight. The active mass loading of NbWO electrode is about 1.5 mg cm^{-2}. We also employed polytetrafluoethylene (PTFE) as the binder to prepare the free-standing films of the working electrodes for the *ex situ* XPS, XRD, and thick electrode measurements. Aluminum and titanium rods were used as negative electrode and positive electrode current collectors, respectively. Filter paper was used as the separator. The electrolyte was purged by N_2 for 30 minutes before use, and the cell was assembled in a simply equipped nitrogen glovebox. Cyclic voltammetry (CV) was carried out on an EC-Lab VMP300 multichannel workstation at various rates. The second cycle of CVs was used for kinetic analysis. Galvanostatic charge-discharge (GCD) measurements were conducted on a LAND CT2001A cell test instrument.

Electrolyte conductivity (σ, S cm^{-1}) of electrolytes was calculated from the electrochemical impedance spectroscopy (EIS) on a Biologic EC-Lab VMP300 with a frequency range of 100 kHz to 100 Hz at 25 °C. The test cell consisted of two parallel titanium rod electrodes on either side of a Φ0.5 × 0.1 cm ($r_{\text{radius}} \times l$) electrolyte reservoir. The uncompensated resistance (R, Ω) from the Nyquist plot was assumed to be dominated by electrolyte resistance and used to calculate conductivity.

$$\sigma = l/R \cdot S \quad \text{(S1)}$$

$$S = \pi r_{\text{radius}}^2 \quad \text{(S2)}$$

The specific energy density (E, Wh kg^{-1}) and power density (P, W kg^{-1}) of the ALIC can be obtained as follows [S2, S3]:

$$E = \int_{t1}^{t2} UIdt \quad \text{(S3)}$$

$$P = E/\Delta t \quad \text{(S4)}$$

$$\Delta t = t_2 - t_1 \quad \text{(S5)}$$

where I is the current density normalized by the total active mass in both electrodes (A g^{-1}), and t_1 and t_2 are the start and end time during the discharge process (h), U is the voltage (V).

S1.4 Molecular Dynamics (MD) Simulations

Molecular dynamics (MD) simulations were performed for 1 m LiAc and 13 m LiAc electrolytes, in order to examine the composition of the hydration shell of cations. Relevant data for the systems can be found in Table S1. The OPLS-AA force field is chosen for its ability to accurately simulate liquid phase systems [S4-S7]. The MD computer simulation process is a combination of three steps. The first step is energy minimization. When packmol [S8] is used to fill a certain percentage of molecules into a cubic box with periodic boundary conditions in all three Cartesian directions, it will produce some unreasonable contact between molecules, and the system energy is in a higher state. Therefore, we pass the first step to minimizing the energy of the system. The conjugate gradient algorithm is used to minimize energy and the tolerance is set to 10 kJ mol^{-1} nm^{-1}. The second step is the equilibrium phase. In order to achieve the lowest energy isomer state of each molecule in the system, we perform a single annealing method in the NVT ensemble. The temperature is maintained by the Velocity-rescale thermostat with a time constant of 0.1 ps [S9]. First, the system is heated to 500 K in 30 ps, then keep the temperature for 10 ps, and finally cooled to 298.15 K in 2 ns. The final step is the production phase. The production simulations are performed in the NPT ensemble at constant pressure and constant temperature. The temperature is maintained by the Velocity-rescale thermostat at 298.15 K for all other systems with a time constant of 0.1 ps. A constant pressure of 1 bar is controlled by the Parrinello–Rahman barostat [S10, S11] with a coupling constant of 2 ps. Electrostatic interactions are treated using the Particle -Mesh-Ewald (PME) method [S12, S13] with the Fourier spacing of 0.118 nm and 0.9 nm real-space cut-off. The visualization of the molecular structures (see Fig. 1) was realized utilizing the non-commercial Visual Molecular Dynamics (VMD) software [S14].

S2 Supplementary Figures

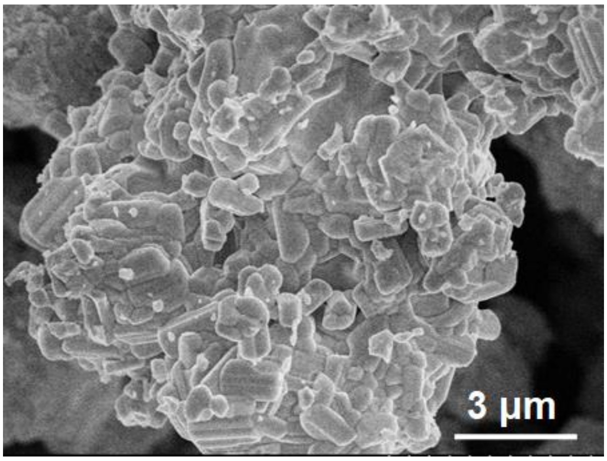

Fig. S1 SEM image of NbWO

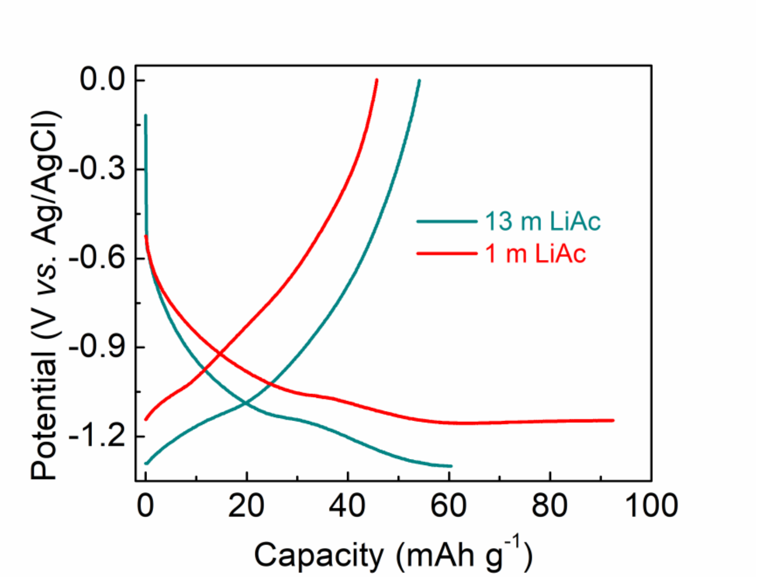

Fig. S2 First GCD curves of NbWO electrode in 1 m and 13 m LiAc electrolytes

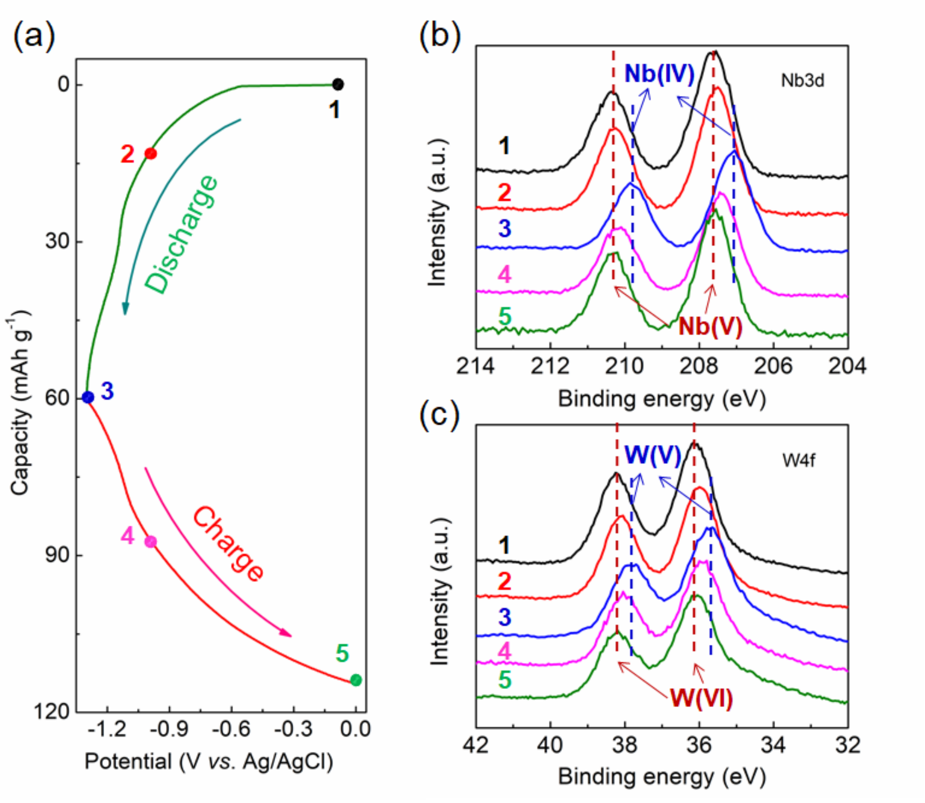

Fig. S3 *Ex situ* XPS spectra of NbWO electrode at different charges of state

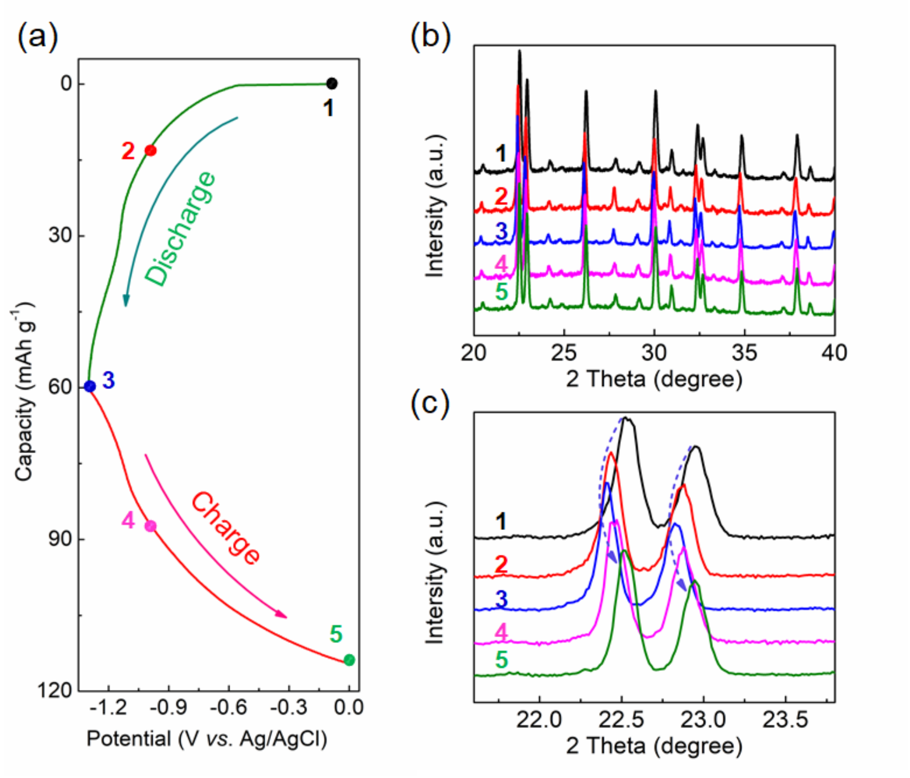

Fig. S4 (**a**) Typical charge/discharge profiles of NbWO for Li-ion storage. (**b**) *Ex situ* XRD patterns of NbWO electrode at selected charge of state in (**a**). (**c**) Angle range of the XRD patterns over the (001) and (190) diffraction peaks

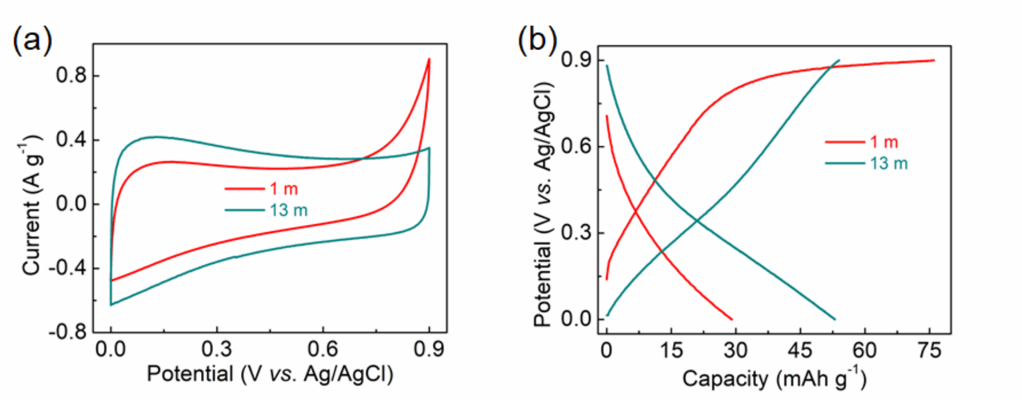

Fig. S5 Electrochemical behaviors of OECG positive electrode in 1 m and 13 m LiAc electrolytes. (**a**) Typical CV curves at 2 mV s^{-1}. (**b**) Typical GCD curve at 200 mA g^{-1}

Obviously, OECG electrode exhibits a reversible adsorption/desorption behavior with Ac^{-} in 13 m LiAc electrolyte with the potential window of 0-0.9 V vs. Ag/AgCl. However, in 1 m LiAc electrolyte, it doesn't work well in the same potential window.

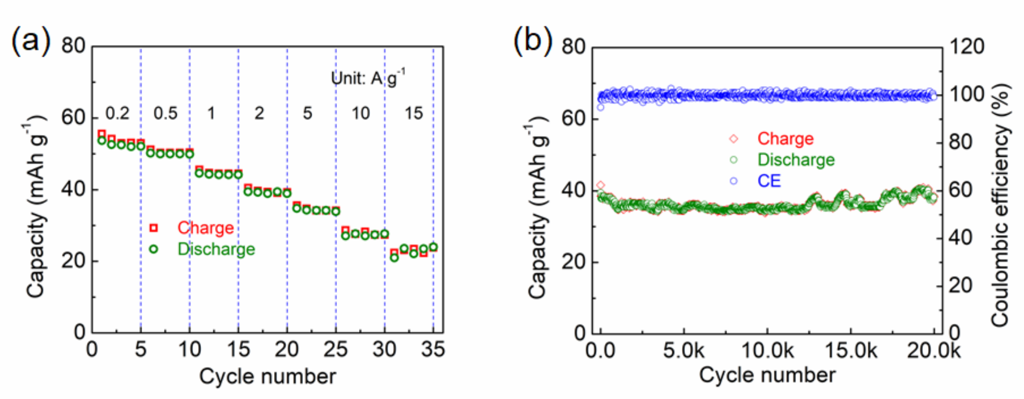

Fig. S6 Electrochemical performance of OECG positive electrode in 13 m LiAc electrolyte. (**a**) Rate performance. (**b**) Cycling stability at 2 A g^{-1}

OECG possesses high reversible capacity of about 53.3 mAh g^{-1} at a current density of 200 mA g^{-1}. The capacity still retains 24.0 mAh g^{-1} even at 15 A g^{-1}. After 20,000 cycles, the capacity retention is about 96% at 2 A g^{-1}.

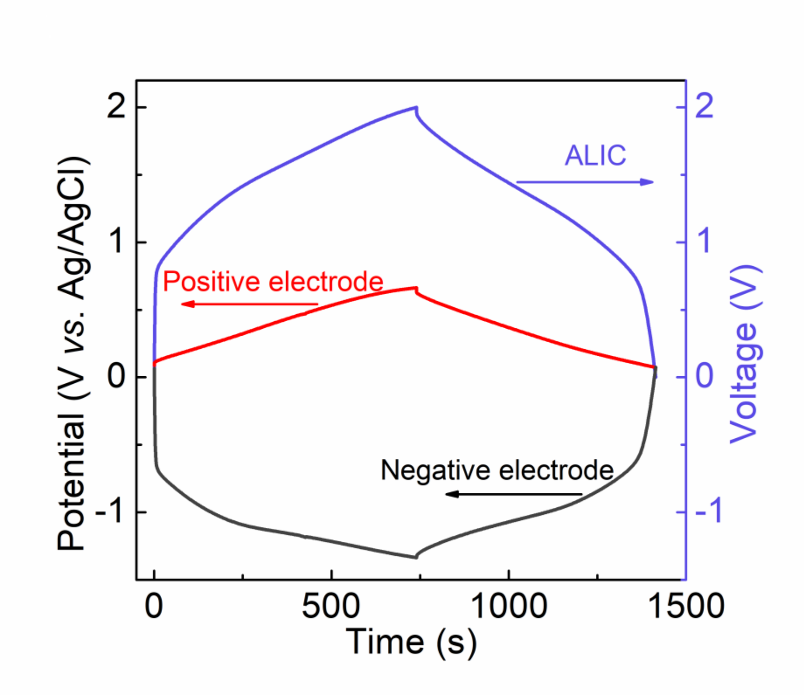

Fig. S7 GCD curves of the negative electrode and positive electrode *vs*. Ag/AgCl reference electrode, along with the voltage profile of the OECG//NbWO ALIC at a current density of 0.2 A g^{-1}

Table S1 Numbers of ions and solvent molecules in the investigated simulation electrolytes

Electrolytes	Li^+	Ac^-	H_2O
1 m LiAc	20	20	1110
13 m LiAc	260	260	1110

Table S2 Physical properties of the electrolytes at 25 °C

Electrolytes	Salt molar fraction (%)	Density from MD (g cm^{-3})	Viscosity from MD (mPa s)	Conductivity (mS cm^{-1})
1 m LiAc	1.8	0.99	0.5	94.6
13 m LiAc	19.0	1.18	9.5	65.5

Table S3 Cycling performance compared with other aqueous and non-aqueous LICs reported in the literatures

References	Negative electrode	Positive electrode	Cycling No.	Capacity retention (100%)
This work	$Nb_{18}W_{16}O_{93}$	OECG	50000	100
[S15]^(aq.)	V_2O_5 nanosheets	Carbon fibers	10000	90
[S16]^(aq.)	AC	CoO@PPy	20000	91.5
[S17]^(aq.)	Grapehen/carbon fiber	Co−Ni−S/carbon fiber	20000	82.2
[S18]	T-Nb_2O_5@C	AC	1000	75
[S19]	Ti_2CT_x	$Na_2Fe_2(SO_4)_3$	100	100
[S20]	TiC	N-doped porous carbon	5000	82
[S21]	NbN	PANI-derived carbon	15000	95
[S22]	Hard carbon	AC	10000	82

^(aq.) Aqueous LICs

Supplementary References

[S1] S. Dong, Y. Xu, L. Wu, H. Dou, X. Zhang, Surface-functionalized graphene-based quasi-solid-state na-ion hybrid capacitors with excellent performance. Energy Storage Mater. **11**, 8-15 (2018). https://doi.org/10.1016/j.ensm.2017.09.006

[S2] H. Wang, C. Zhu, D. Chao, Q. Yan, H. Fan, Nonaqueous hybrid lithium-ion and sodium-ion capacitors. Adv. Mater. **29**(46), 1702093, (2017). https://doi.org/10.1002/adma.201702093

[S3] S. Dong, Z. Li, Z. Xing, X. Wu, X. Ji, X. Zhang, Novel potassium-ion hybrid capacitor based on an anode of $K_2Ti_6O_{13}$ microscaffolds. ACS Appl. Mater. Interfaces **10**(18), 15542-15547 (2018). https://doi.org/10.1021/acsami.7b15314

[S4] W.L. Jorgensen, D.S. Maxwell, J. Tirado-Rives, Development and testing of the opls all-atom force field on conformational energetics and properties of organic liquids. J. Am. Chem. Soc. **118**(45), 11225-11236 (1996). https://doi.org/10.1021/ja9621760

[S5] D. Bedrov, O. Borodin, G.D. Smith, Molecular dynamics simulation of 1,2-dimethoxyethane/water solutions. 2. Dynamical properties. J. Phys. Chem. B **102**(47), 9565-9570 (1998). https://doi.org/10.1021/jp982161j

[S6] J-C. Soetens, C. Millot, B. Maigret, Molecular dynamics simulation of Li+BF4- in ethylene carbonate, propylene carbonate, and dimethyl carbonate solvents. J. Phys. Chem. A **102**(7), 1055-1061 (1998). https://doi.org/10.1021/jp972457

[S7] J. Wang, R.M. Wolf, J.W. Caldwell, P.A. Kollman, D.A. Case, Development and testing of a general amber force field. J. Comput. Chem. **25**(9), 1157-1174 (2004). https://doi.org/10.1002/jcc.20035

[S8] L. Martínez, R. Andrade, E.G. Birgin, J.M. Martínez, Packmol: A package for building initial configurations for molecular dynamics simulations. J. Comput. Chem. **30**(13), 2157-2164 (2009). https://doi.org/10.1002/jcc.21224

[S9] G. Bussi, D. Donadio, M. Parrinello, Canonical sampling through velocity rescaling. J. Chem. Phys. **126**(1), 014101 (2007). https://doi.org/10.1063/1.2408420

[S10] M. Parrinello, A. Rahman, Crystal structure and pair potentials: A molecular-dynamics study. Phys. Rev. Lett. **45**(14), 1196-1199 (1980). https://doi.org/10.1103/PhysRevLett.45.1196

[S11] M. Parrinello, A. Rahman, Polymorphic transitions in single crystals: A new molecular dynamics method. J. Appl. Phys. **52**(12), 7182-7190 (1981). https://doi.org/10.1063/1.328693

[S12] T. Darden, D. York, L. Pedersen, Particle mesh ewald: An N·log(N) method for ewald sums in large systems. J. Chem. Phys. **98**(12), 10089-10092 (1993). https://doi.org/10.1063/1.464397

[S13] U. Essmann, L. Perera, M.L. Berkowitz, T. Darden, H. Lee, L.G. Pedersen, A smooth particle mesh ewald method. J. Chem. Phys. **103**(19), 8577-8593 (1995). https://doi.org/10.1063/1.470117

[S14] W. Humphrey, A. Dalke, K. Schulten, Vmd: Visual molecular dynamics. J. Mol. Graph. **14**(1), 33-38 (1996). https://doi.org/10.1016/0263-7855(96)00018-5

[S15] L. Li, S. Peng, H.B. Wu, L. Yu, S. Madhavi, X.W. Lou, A flexible quasi-solid-state asymmetric electrochemical capacitor based on hierarchical porous V_2O_5 nanosheets on carbon nanofibers. Adv. Energy Mater. **5**(17), 1500753 (2015). https://doi.org/10.1002/aenm.201500753

[S16] C. Zhou, Y. Zhang, Y. Li, J. Liu, Construction of high-capacitance 3D CoO@polypyrrole nanowire array electrode for aqueous asymmetric supercapacitor. Nano Lett. **13**(5), 2078-2085 (2013). https://doi.org/10.1021/nl400378j

[S17] W. Chen, C. Xia, H.N. Alshareef, One-step electrodeposited nickel cobalt sulfide nanosheet arrays for high-performance asymmetric supercapacitors. ACS Nano **8**(9), 9531-9541 (2014). https://doi.org/10.1021/nn503814y

[S18] E. Lim, C. Jo, H. Kim, M-H. Kim, Y. Mun et al., Facile synthesis of Nb_2O_5@carbon core-shell nanocrystals with controlled crystalline structure for high-power anodes in hybrid supercapacitors. ACS Nano **9**(7), 7497-7505 (2015). https://doi.org/10.1021/acsnano.5b02601

[S19] X. Wang, S. Kajiyama, H. Iinuma, E. Hosono, S. Oro, I. Moriguchi, M. Okubo, A. Yamada, Pseudocapacitance of mxene nanosheets for high-power sodium-ion hybrid capacitors. Nat. Commun. **6**, 6544 (2015). https://doi.org/10.1038/ncomms7544

[S20] H. Wang, Y. Zhang, H. Ang, Y. Zhang, H.T. Tan et al., A high-energy lithium-ion capacitor by integration of a 3D interconnected titanium carbide nanoparticle chain anode with a pyridine-derived porous nitrogen-doped carbon cathode. Adv. Funct. Mater. **26**(18), 3082-3093 (2016). https://doi.org/10.1002/adfm.201505240

[S21] P. Wang, R. Wang, J. Lang, X. Zhang, Z. Chen, X. Yan, Porous niobium nitride as a capacitive anode material for advanced li-ion hybrid capacitors with superior cycling stability. J. Mater. Chem. A **4**(25), 9760-9766 (2016). https://doi.org/10.1039/c6ta02971j

[S22] J.H. Kim, J-S. Kim, Y-G. Lim, J-G. Lee, Y-J. Kim, Effect of carbon types on the electrochemical properties of negative electrodes for Li-ion capacitors. J. Power Sources **196**(23), 10490-10495 (2011). https://doi:10.1016/j.jpowsour.2011.08.081
